# The ion balance of Shotokuseki extract promotes filaggrin fragmentation and increases amino acid production and pyrrolidone carboxylic acid content in three-dimensional cultured human epidermis

**DOI:** 10.1007/s13659-022-00353-0

**Published:** 2022-10-17

**Authors:** Kei Tsukui, Takuya Kakiuchi, Masamitsu Suzuki, Hidetomo Sakurai, Yoshihiro Tokudome

**Affiliations:** 1 Laboratory of Cosmetic Sciences, Graduate School of Advanced Health Sciences, Saga University, 1 Honjo, Saga, 840-8502 Japan; 2 Laboratory of Dermatological Physiology, Faculty of Pharmacy and Pharmaceutical Sciences, Josai University, 1-1 Keyakidai, Sakado, Saitama 350-0295 Japan; 3 Zeria Pharmaceutical Co., Ltd., 10-11 Nihonbashi, Kobuna-cho, Chuo-ku, Tokyo, 103-8351 Japan; 4 Laboratory of Cosmetic Sciences, Regional Innovation Center, Saga University, 1 Honjo, Saga, 840-8502 Japan

**Keywords:** Shotokuseki extract, Epidermis, Amino acid, Pyrrolidone carboxylic acid, Ion, Gene expression

## Abstract

Natural moisturizing factor (NMF) in the stratum corneum contributes to the retention of moisture there. The purpose of this study was to determine the penetration of ions in Shotokuseki extract (SE) into the three-dimensional cultured epidermis and the effect of NMF on the biosynthesis of amino acids and pyrrolidone carboxylic acid formation. Various ions, amino acids and pyrrolidone carboxylic acid were quantified by inductively coupled plasma mass spectrometry, fully automatic amino acid analyzer or high-performance liquid chromatography (HPLC) in three-dimensional cultured epidermis after application of SE. Gene expression levels of profilaggrin, calpain1, caspase14, and bleomycin hydrolase, which are involved in NMF production, were determined by reverse-transcription qPCR and bleomycin hydrolase activity was determined by aminopeptidase assay. The application of SE increased Na, K, Mg, Ca, Al, and Fe levels in three-dimensional cultured epidermis. The mRNA levels of the starting material of amino acid synthesis profilaggrin, and calpain1 and bleomycin hydrolase, which are involved in its fragmentation, increased. The activity of bleomycin hydrolase also increased. Furthermore, the levels of amino acids and pyrrolidone carboxylic acid increased in the three-dimensional cultured epidermis. This suggests that the ionic composition of SE may be involved in its moisturizing effect on the stratum corneum.

**Graphical Abstract:**

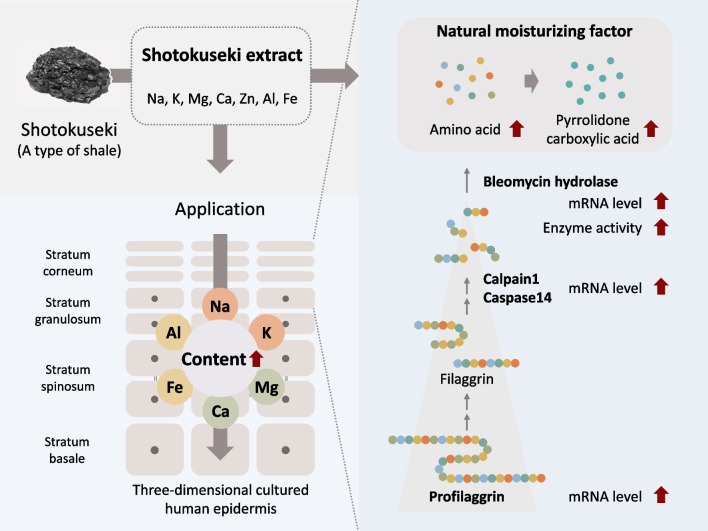

## Introduction

The stratum corneum is the outermost layer of the epidermis. It has an important role in maintaining skin moisture and protecting the skin from the external environment. Natural moisturizing factor (NMF) is a major factor in maintaining skin moisture [1, 2]. It is present in keratinocytes and contributes to the epidermal barrier function and moisture retention function [2]. It is generally composed of amino acids (40%), pyrrolidone carboxylic acid (PCA, 12%), lactate (12%), and urea (7%) [3]. NMF is synthesized from filaggrin (FLG) in the stratum corneum [4–7]. FLG is produced by the precursor protein profilaggrin (proFLG) within the keratohyalin granules [8]. proFLG is metabolized to FLG by dephosphorylation and other mechanisms during epidermal differentiation, and associates with keratin in keratinocytes [9]. In the upper stratum corneum, proteolytic enzymes such as Calpain1 (CAPN1) and Caspase14 (CASP14) degrade FLG from keratin [10–14]. Arginine in FLG is citrullinated (Cit) by protein arginine deiminase, making it more susceptible to proteolytic enzymes. Finally, Cit residues of FLG are released and fragmented into amino acids by bleomycin hydrolase (BH) [15, 16]. The amino acids Glu and Gln are converted to PCA by γ-glutamyltransferase [17]. His is converted to urocaninic acid by histase, and Arg is converted to urea by arginase [18, 19].

NMF is a small water-soluble molecule that is involved in the moisturizing function of the stratum corneum. The content of free amino acids in the stratum corneum of patients with dry skin, such as in ichthyosis, psoriasis, and senile xeroderma, is significantly reduced [3, 20–23]. In atopic dermatitis (AD), BH expression is reduced in both lesion and non-lesion sites [24]. Quantitative and qualitative changes in amino acids in the stratum corneum are considered a major factor in dry skin. In addition, PCA content is decreased in the skin of AD patients, especially in the lesion area [25]. PCA content has also been reported to correlate with skin barrier function [26].

Various ions have the following effects on the epidermis: Na, water retention [27]; K, improved epidermal barrier function [28]; Mg, improved epidermal barrier function [29] and anti-inflammatory activity [30]; Ca, promotes epidermal differentiation [31–33] and regulates epidermal hyaluronic acid synthesis [34]; and Zn, promotes epidermal cell growth [35], and exerts antioxidant [35] and anti-inflammatory effects [36]. Ionic components derived from natural products, such as hot spring water and deep sea water have long been used for cosmetic and other purposes.

Shotokuseki is a type of rock formed by the precipitation of plankton and seaweed. The water extract of Shotokuseki is called Shotokuseki extract (SE), which is used for cosmetics. However, the effects of SE on the skin and its mechanism of action are not well understood. We previously reported that Na, K, Mg, Ca, and Zn are present in SE. We also reported that SE applied to keratinocytes increased the expression of an epidermal differentiation marker and increased intracellular calcium concentration compared with application of the same concentration of calcium [37]. In this study, the ions in SE were further analyzed. We also examined the effect of the production of amino acids and pyrrolidone carboxylic acid in three-dimensional cultured human epidermis.

## Results

### Measurement of ion content in SE

**Fig. 1:**
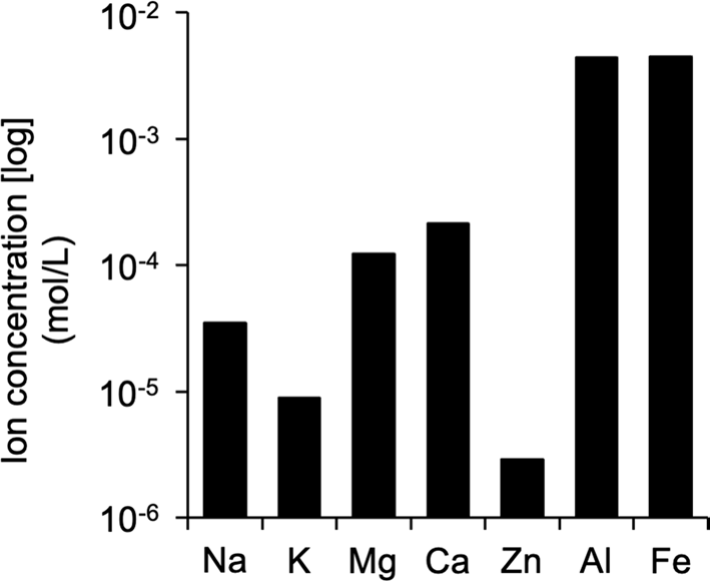
Ion concentration of SE. Mineral concentration was analyzed by inductively coupled plasma/mass spectrometry (ICP/MS). Data are expressed as independent experiments. Values were averages of two measurements of the sample

Ion content in SE was measured by inductively coupled plasma mass spectrometry (ICP/MS). The concentrations of Na, K, Mg, Ca, Zn, Al, and Fe ions were 0.035, 0.009, 0.123, 0.212, 0.003, 4.448, and 4.476 mM, respectively (Fig. 1).

### Ion content of three-dimensional cultured human epidermis after SE application

**Fig. 2:**
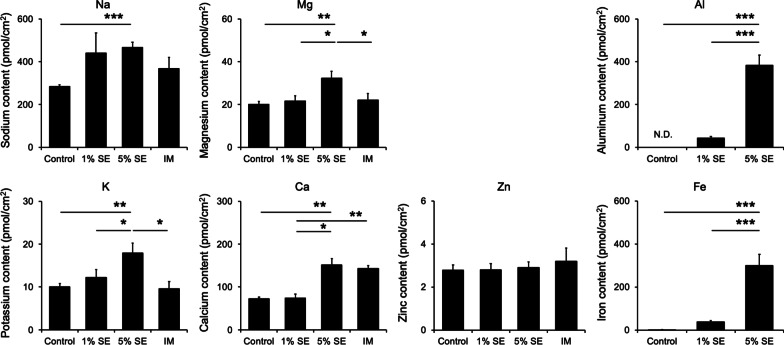
Ion content in three-dimensional cultured human epidermis after application of SE. Three-dimensional cultured human epidermis was treated with SE or IM for 8 days. Data are expressed as mean ± S.D. of three independent experiments. *p < 0.05, **p < 0.01, and ***p < 0.001. Statistical significance was evaluated using Tukey’s post hoc multiple comparison test. *N.D.* not detected

The ion content of three-dimensional cultured human epidermis after the application of SE was determined by ICP/MS analysis. At the same time, a mixture of the five ions contained in SE (ion mixture; IM) was also examined after application. IM contained Na, K, Mg, Ca, and Zn, equal to the concentrations of each ion in SE. Na content in the epidermis increased in the 5% SE group compared with that in the control group. The contents of K and Mg in the epidermis also increased in the 5% SE group compared with the levels in the Control, 1% SE, and IM groups. Moreover, epidermal Ca content increased in the 5% SE group compared with those in the Control and 1% SE groups. However, there was no difference in this regard between the 5% SE and IM groups. Epidermal Zn content was similar in all groups. The contents of Al and Fe in the epidermis increased in the 5% SE group compared with those in the Control and 1% SE groups. Finally, Al was not detected in the epidermis of the Control group (Fig. 2).

### Effects of NMF-related gene expression in three-dimensional cultured human epidermis after SE application

**Fig. 3:**
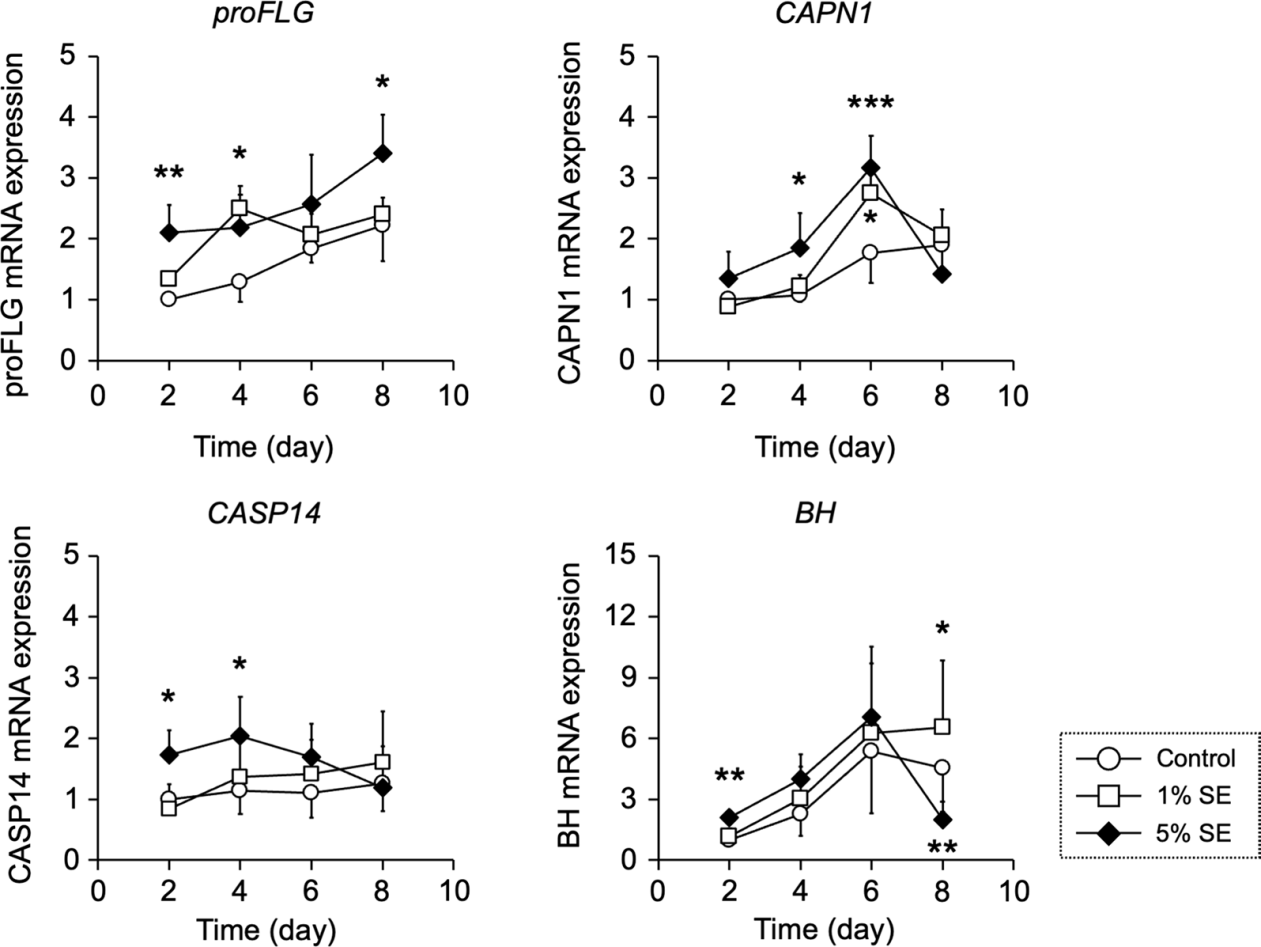
Effects of natural moisturizing factor-related gene expression in three-dimensional cultured human epidermis. Three-dimensional cultured human epidermis was treated with SE for 2–8 days. *CAPN1*, *CASP14*, and *BH* mRNA expression was assessed by quantitative real-time PCR. Data are expressed as mean ± S.D. of four independent experiments. *p < 0.05, **p < 0.01, and ***p < 0.001 (compared with control group at the same time point). Statistical significance was evaluated using Dunnett’s test

The expression levels of genes related to NMF production and metabolism were measured by real-time PCR. The mRNA expression levels of proFLG, CAPN1, CASP14, and BH increased 3.4-, 3.2-, 2.0-, and 7.1-fold, respectively, upon the application of 5% SE (Fig. 3).

### Effects of bleomycin hydrolase activity in three-dimensional cultured human epidermis after SE application

**Fig. 4:**
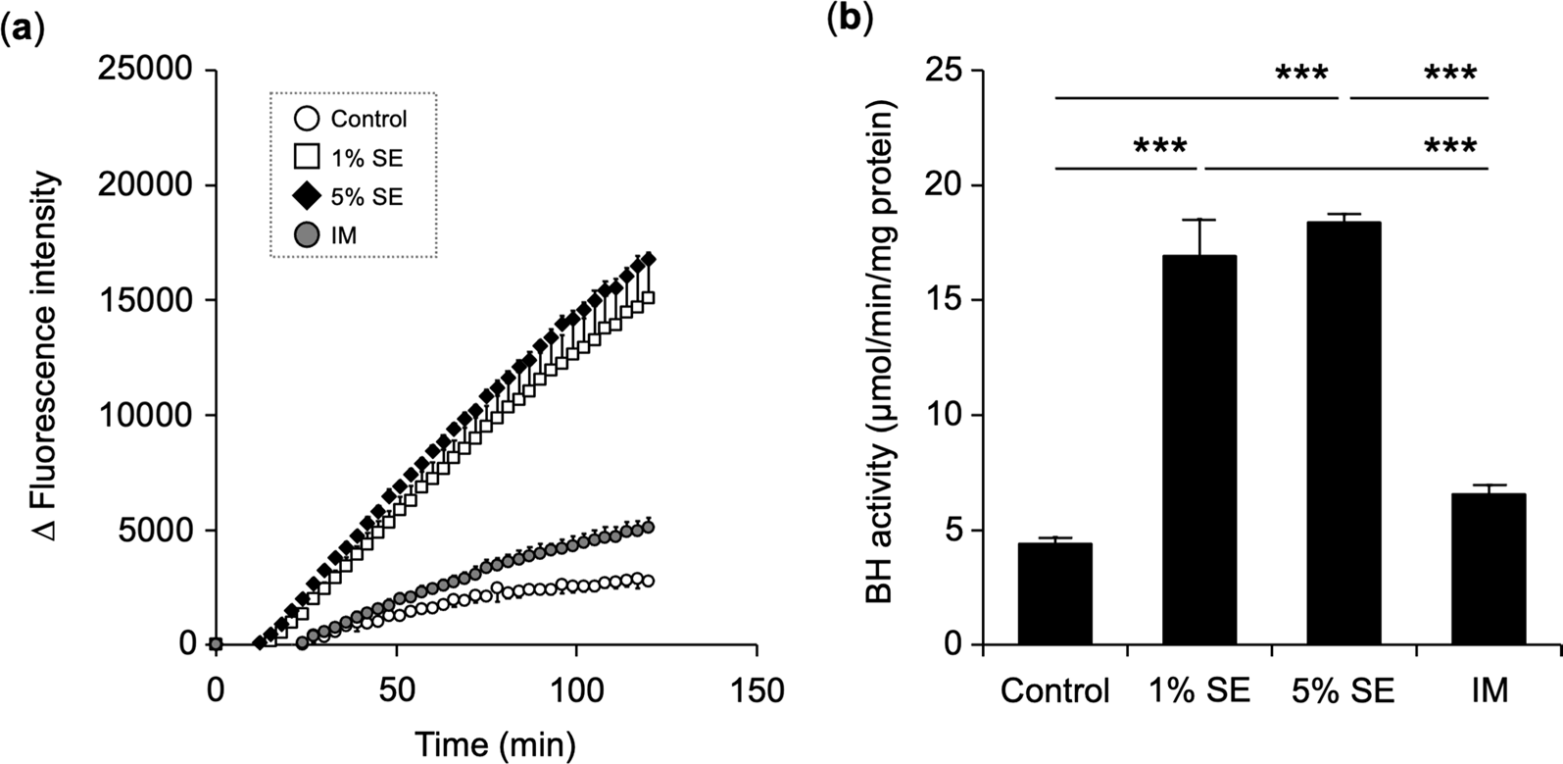
Effects of BH activity in three-dimensional cultured human epidermis. Three-dimensional cultured human epidermis was treated with SE or IM for 8 days. Samples were assayed for BH activity toward citrulline-4-methylcoumaryl-7-amide (Cit-AMC). **a** Amount of change in fluorescence intensity up to 120 min. **b** BH activity. Data are expressed as mean ± S.D. of three independent experiments. ***p < 0.001. Statistical significance was evaluated using Tukey’s post hoc multiple comparison test

The BH activity of three-dimensional cultured human epidermis after SE application was measured by aminopeptidase assay. BH releases Cit and produces 7-amino-4-methylcoumarin (AMC). SE application increased AMC production. SE also resulted in a significant increase of aminopeptidase activity against Cit-AMC fluorescent substrate (Fig. 4a). BH activity increased 3.8-fold and 4.2-fold in the 1% SE group and 5% SE group, respectively, compared with that in the control group (Fig. 4b).

### Effects on amino acid and pyrrolidone carboxylic acid content in three-dimensional cultured human epidermis after SE application

**Fig. 5:**
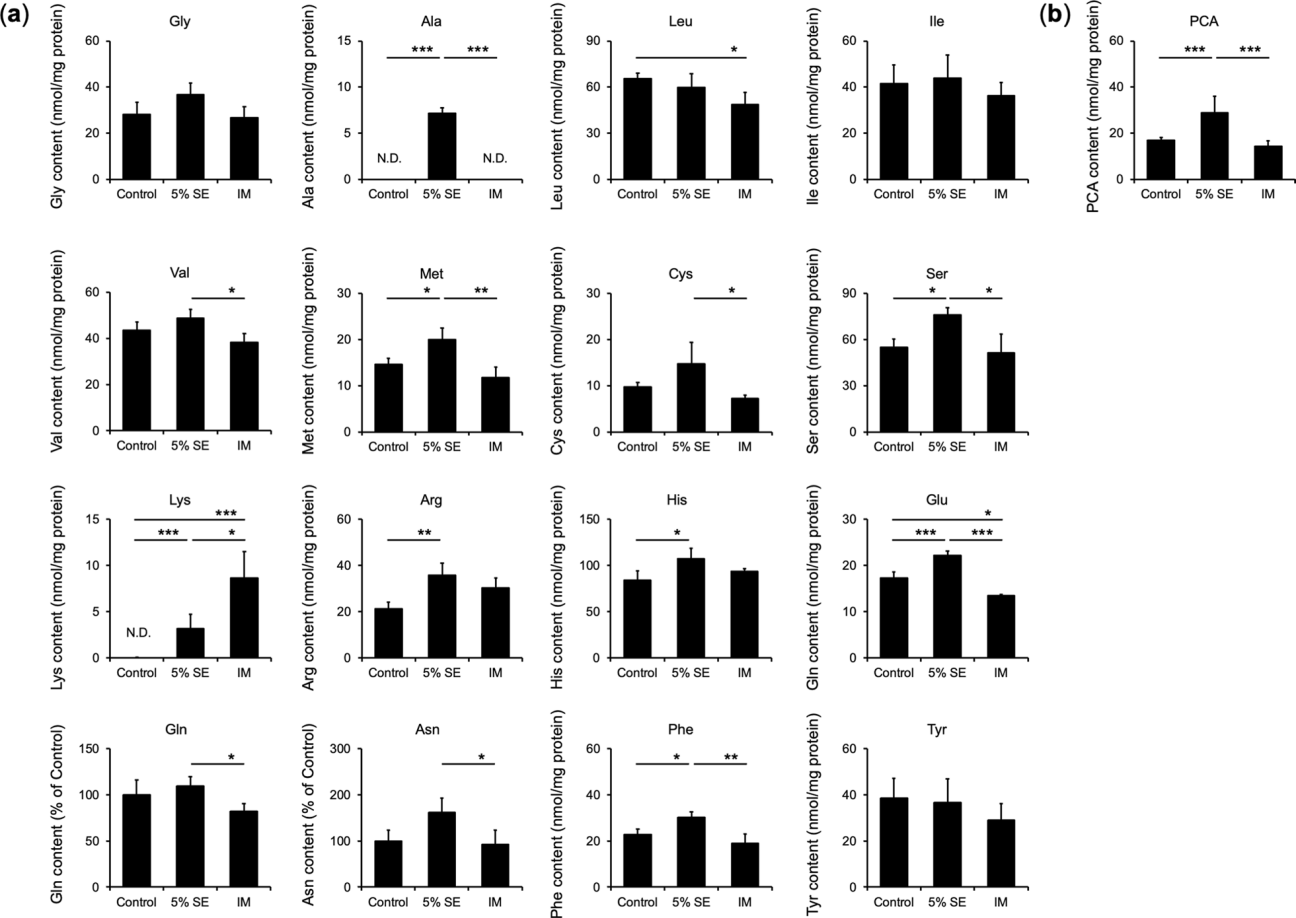
Effects of natural moisturizing factor content in three-dimensional cultured human epidermis. Three-dimensional cultured human epidermis was treated with SE or IM for 8 days. **a** Amino acid content, **b** PCA content. **a** Amount of amino acids was quantified by a fully automatic amino acid analyzer. Data are expressed as mean ± S.D. of four independent experiments. *p < 0.05, **p < 0.01, and ***p < 0.001. Statistical significance was evaluated using Tukey’s post hoc multiple comparison test. *N.D.* not detected. **b** Amount of PCA was quantified by high-performance liquid chromatography (HPLC) analysis. Data are expressed as mean ± S.D. of four independent experiments. *p < 0.05 and **p < 0.01. Statistical significance was evaluated using Tukey’s post hoc multiple comparison test

The levels of eight amino acids (Ala, Met, Ser, Lys, Arg, His, Glu, and Phe) in the epidermis were increased in the 5% SE group compared with those in the control group. In addition, Ala, Val, Met, Cys, Ser, Lys, Glu, Gln, Asn, and Phe increased compared with the levels in the IM group. PCA in three-dimensional cultured human epidermis after SE application was also quantified. The amount of PCA in the epidermis increased 1.7-fold in the 5% SE group compared with that in the control group and 2.0-fold compared with that in the IM group (Fig. 5).

## Discussion

The water extract of Shotokuseki is called Shotokuseki extract (SE), which is used for cosmetics. However, the effects of SE on the skin and its mechanism of action are not well understood. We previously reported that Na, K, Mg, Ca, and Zn are present in SE. We also reported that SE applied to keratinocytes increased the expression of an epidermal differentiation marker and increased intracellular calcium concentration compared with application of the same concentration of calcium [37]. In this study, the ions in SE were further analyzed. We also examined the effect of the production of amino acids and pyrrolidone carboxylic acid in three-dimensional cultured human epidermis. This study confirmed the effects of SE on the production of amino acids and pyrrolidone carboxylic acid and considered SE’s potential as a humectant. The penetration of ions in SE into the epidermis was also examined. by ICP/MS. The results showed that Fe and Al concentrations were 4.5 and 4.4 mM, respectively (Fig. 1). When considering the effects of various ions on the skin, it is necessary to confirm that the target ion is actually delivered into the skin. Although polar compounds such as ions are considered to have low skin permeability [38], in this study we confirmed that various ions applied from the stratum corneum side of three-dimensional cultured human epidermis penetrated the epidermis (Fig. 2). These ions that permeated the viable epidermis are expected to have bioactive effects on the skin. It is usually known that ions and other substances do not easily penetrate the skin. However, three-dimensional cultured human epidermis is generally considered to have a weaker barrier function than human skin tissue. Moreover, the living epidermal layer under the stratum corneum contains transporters for various ions such as Na, K, Mg, and Ca [39–42]. Further investigation, it will be necessary to evaluate the penetration of ions in human skin.

We previously reported that SE promotes keratinocyte differentiation [37]. Corneocytes are differentiated keratinocytes that comprise the outermost layer of the epidermis. NMF is located in the corneocytes and is an important factor for the retention of water in the stratum corneum. We hypothesized that the promotion of keratinocyte differentiation by SE affected NMF production. The NMF content in the epidermis was measured after SE application. NMF is produced by filaggrin fragmentation [4, 5]. The amino acids that comprise filaggrin include Ser, Gln, Glu, Gly, Arg, and His, in decreasing order of abundance. NMF component amino acids are similarly abundant in this order. The amounts of amino acids in the epidermis after SE application increased for Ala, Met, Ser, Lys, Arg, His, Glu, and Phe. (Fig. 5a). The amino acids increased by SE application were similar to the amino acid sequence of FLG. Previous studies have also quantified amino acids in human epidermis and reported that amino acids such as Ser, Ala, Gly, and His [43, 44]. This result suggests that the application of SE promoted the fragmentation of FLG and increased its amino acid content. Given that Glu content in the epidermis was increased, the PCA content of its metabolites was measured. The results showed that the PCA content of the epidermis increased similarly to the Glu content. Gln and Glu are metabolized by γ-glutamyltransferase to PCA [17]. These findings support the assertion that the addition of SE increases PCA (Fig. 5b). The relationship between the expression of FLG fragment proteins in the stratum corneum and PCA contents has been reported that in skin with reduced CASP14 or BH protein levels, PCA contents are also reduced [13, 45, 46]. To corroborate that the application of SE increases amino acid and PCA content, mRNA expression of NMF precursor proFLG was measured. The results showed that mRNA expression of the precursor proFLG was increased as well as NMF levels (Fig. 3). Next, mRNA expression levels of CAPN1, CASP14, and BH, which are involved in filaggrin fragmentation, were measured in association with the increased NMF content in the epidermis. The results showed that the application of SE increased these levels (Fig. 3). The activity of BH involved in the final step of NMF generation was also evaluated, with SE application being shown to significantly increase the activity of BH. In contrast, treatment with IM containing the same concentrations of Na, K, Mg, Ca, and Zn as SE did not increase BH (Fig. 4). Ions other than Na, K, Mg, Ca, and Zn may be involved in epidermal BH activity and increase NMF levels secondary to enhanced filaggrin fragmentation. Choi et al. reported that application of the ceramide metabolite phytosphingosine increased CASP14 and BH expression and PCA content [46]. In the future, it will be necessary to elucidate which ions and other substances in SE are responsible for these effects. Whether there are differences between the actions of SE and IM due to the interaction of various ions should be investigated in more detail in future work due to the action of biological trace ions. The interaction of various ions and the action of biological trace ions must also be considered. In addition to NMF, intercellular lipids in the stratum corneum are important factors for the barrier function of the stratum corneum. We thus think that investigating the effects of SE on the production and metabolism of intercellular lipids in the stratum corneum will provide clues to understanding their effects on skin barrier function.

## Experimental section

### Materials

SE was provided by IONA International Corporation (Tokyo, Japan), a member of the Zeria Group (Tokyo, Japan). LabCyte EPI-MODEL 6D (three-dimensional cultured human epidermis) and culture medium were purchased from Japan Tissue Engineering (Gamagori, Aichi, Japan). AMC (7-Amino-4-methylcoumarin) was purchased from Sigma-Aldrich (St. Louis, MO, USA). Citrulline 7-amino-4-methylcoumarin (H-Cit-AMC) was purchased from Bachem AG (Bubendorf, Basel, Switzerland). Primers were purchased from Thermo Fisher Scientific (Waltham, MA, USA). RNAiso Plus, PrimeScript™ RT Reagent Kit, and TB Green™ Premix ExTaq were purchased from TaKaRa Bio Inc. (Kusatsu, Shiga, Japan). XSTC-1, 7, 8, and 13 were purchased from SPEX (Metuchen, NJ, USA). Lithium citrate buffers (1st to 5th) were purchased from JEOL (Tokyo, Japan). All other chemicals and solvents used were of analytical grade and purchased from FUJIFILM Wako Pure Chemical Corporation (Osaka, Japan).

### General experimental procedures

#### Preparation of Shotokuseki extract

To prepare Shotokuseki extract, purified water was added to Shotokuseki (a type of shale), and after a managed period of time, the Shotokuseki was removed. The weight ratio of purified water and Shotokuseki was 50:1.

#### Inductively coupled plasma/mass spectrometry (ICP/MS)

The ion content of SE and three-dimensional cultured human epidermis was determined by inductively coupled plasma mass spectrometry (ICP/MS, NexION350S; PerkinElmer, Inc., Waltham, MA, USA). Three-dimensional cultured human epidermis was immersed in nitric acid and hydrogen peroxide. After heat dissolution, ions were measured by dilution with pure water containing 38% hydrofluoric acid. Calibration curves were prepared using XSTC-1, 7, 8, and 13.

#### Preparation of ion mixture solution

The ion mixture solution (IM) was prepared using Na_2_SO_4_, MgSO_4_, K_2_SO_4_, CaSO_4_, ZnSO_4_, and H_2_SO_4_ (at pH 1.6). The solution contained 0.03 mM Na, 0.01 mM K, 0.14 mM Mg, 0.21 mM Ca, and 0.004 mM Zn, equal to the concentrations of each ion in SE as measured by ICP/MS.

#### Cell culture

Three-dimensional cultured human epidermis model were pre-incubated for 3 h and then treated with the indicated solution every day for 2–8 days from the stratum corneum side. The skin models were cultured in culture medium provided by the manufacturer. The culture medium was changed every day. Three-dimensional cultured human epidermis model were incubated in a humidified atmosphere of 5% CO_2_ at 37 °C.

#### RNA extraction and reverse-transcription real-time PCR (RT-qPCR)

The total RNA was extracted from three-dimensional cultured human epidermis using RNAiso Plus, following the manufacturer’s protocol. RNA purity and concentration were determined using NanoDrop 1000 (Thermo Fisher Scientific). cDNA synthesis was performed using PrimeScript™ RT Reagent Kit, in accordance with the manufacturer’s instructions. The reaction was performed with a thermal cycler (Veriti™ 96 Well; Applied Biosystems, Foster City, CA, USA). The cDNA was applied as a template for qRT-PCR in a Step One Plus Real-Time PCR system (Applied Biosystems) with TB Green™ Premix ExTaq using the following settings: 40 cycles of 95 °C for 5 s and 60 °C for 30 s. Relative expression of the target genes was calculated using the ΔΔCt method [47]. Gene expression was evaluated with primers, including proFLG, 5′-CCATCATGGATCTGCGTGG-3′ (forward) and 5′-CACGAGAGGAAGTCTCTGCGT-3′ (reverse); CAPN1, 5′-CAAACACCCCTCCCCCAGGATGT-3′ (forward) and 5′-CGCACCCGCAGCTGCTCATA-3′ (reverse); CASP14, 5′-TGCACGTTTATTCCACGGTA-3′ (forward) and 5′-TGCTTTGGATTTCAGGGTTC-3′ (reverse); BH, 5′-GTACTTGTGCTGGGGCCTAG-3′ (forward) and 5′-TGGCCCCATAACACCCTTGG-3′ (reverse); and GAPDH, 5′-GAAGGTGAAGGTCGGAGT-3′ (forward) and 5′-GAAGATGGTGATGGGATTTC-3′ (reverse).

#### Bleomycin hydrolase activity assay

BH activity was measured with reference to the method of Kamata et al. [15]. Three-dimensional cultured human epidermis model were immersed in extract solution [10 mM Tris–HCl, 14 mM NaCl, 0.1% (w/v) Tween 20] and lysate was prepared with an ultrasonic homogenizer (SONIFIER 250 Advanced; BRANSON, Danbury, CT, USA). BH activity in the extract was measured at 37 °C for 120 min with extracts and 0.1 mM citrulline 7-amino-4-methylcoumarin (AMC) as a substrate in 0.1 mM Tris–HCl, pH 7.5, containing 10 mM dithiothreitol and 5 mM EDTA. The fluorescence intensity was measured at 360/440 nm using a plate reader (SpectraMax M2^e^; Molecular Devices, Sunnyvale, CA, USA).

#### Extraction of amino acids and PCA from three-dimensional cultured human epidermis

Three-dimensional cultured human epidermis were immersed in extract solution (PBS containing 2% propylene glycol and 0.1% SDS). The solution was homogenized by an ultrasonic homogenizer (SONIFIER 250 Advanced) and centrifuged, after which the supernatant was recovered and the protein concentration was measured by the Lowry method [48].

#### Amino acid analysis

The solution was mixed with an equal volume of 5% 5-sulfosalicylic acid dihydrate, centrifuged, and the supernatant was transferred to an HPLC vial. The amount of amino acids was measured by a fully automatic amino acid analyzer (JLC-500/V; JEOL Ltd.). An LCR-7 (4.0 × 75 mm; JEOL Ltd.) column was used as the pre-column and separation was performed on an ion-exchange column packed with sulfonated styrene–divinylbenzene copolymer LCR-6 (4.0 × 120 mm; JEOL Ltd.) using a stepwise gradient of lithium citrate buffers (1st to 5th) at a flow rate of 0.88 mL/min during chromatographic separation. Detection was carried out at two wavelengths (440 or 570 nm) in accordance with the manufacturer’s instructions. Amino acid standards (Amino Acids Mixture Standard Solution, Type H; FUJIFILM Wako Pure Chemical Corporation) were used for calibration of the instrument, and post-column amino acid derivatization was used for quantitative assessment of amino acids in the eluate, which occurred by mixing the eluate with ninhydrin reagent.

#### Pyrrolidone carboxylic acid analysis by HPLC

The solution was then transferred to HPLC vials for injection onto an HPLC system. The HPLC system consisted of an LC-20AD solvent delivery unit (Shimadzu Corporation, Kyoto, Japan) equipped with an SIL-20AC autosampler (Shimadzu Corporation) and an SPD-20AV UV detector (Shimadzu Corporation) set at a wavelength of 206 nm. Samples were injected onto a SHISEIDO CAPCELL PAK C18 (particle size 5 µm, 250 mm × 4.6 mm; SHISEIDO, Tokyo, Japan) and eluted at 1 mL/min with a mobile phase comprising buffer:acetonitrile at a ratio of 98:2. The buffer was prepared from 10 mM KH_2_PO_4_ and H_3_PO_4_ at pH 2.5.

#### Statistical analysis

Data are presented in this paper as mean ± standard deviation. Statistical analysis among three or more groups was performed by Tukey’s post hoc multiple comparison test, using JMP (version 15.0.0; SAS Institute Inc., Cary, NC, USA).
